# Gastrointestinal Bleeding as an Atypical Presentation of Neonatal Gastric Perforation in a Preterm Infant: A Case Report

**DOI:** 10.7759/cureus.113108

**Published:** 2026-07-21

**Authors:** Ali Naser, Fatima Alameen, Noora Abuzeyad, Eman Shajira, Abdulrahman Alshafei

**Affiliations:** 1 Department of Pediatric Surgery, Bahrain Defense Hospital, Riffa, BHR; 2 Department of Pediatric Surgery, Military Hospital, Royal Medical Services, Riffa, BHR; 3 Department of Orthopedic Surgery, Royal Medical Services, Kingdom of Bahrain, Bahrain, BHR; 4 Department of Pediatrics, Military Hospital, Royal Medical Services, Riffa, BHR

**Keywords:** emergency surgical care, neonatal gastric perforation, neonatal mortality, pneumo peritoneum, preterm neonate

## Abstract

Neonatal gastric perforation is a rare but life-threatening surgical emergency that typically occurs during the first week of life. Early diagnosis remains challenging because the initial clinical and radiographic findings may be nonspecific, particularly in preterm infants, yet prompt recognition and surgical intervention are essential for improving outcomes. We report the case of a four-day-old preterm, low birth weight female infant admitted to the neonatal intensive care unit with prematurity and respiratory distress. During hospitalization, she developed hematemesis and melena. Initial abdominal radiography showed no evidence of pneumoperitoneum despite clinical concerns. Repeated clinical evaluation raised suspicion of gastrointestinal perforation, prompting emergency exploratory laparotomy. Intraoperatively, a gastric perforation was identified and surgically repaired. The postoperative course was complicated by a gastric leak, which required further management. The infant subsequently recovered and was discharged in stable condition. This case highlights the importance of maintaining a high index of suspicion for neonatal gastric perforation in preterm and low-birth-weight infants presenting with gastrointestinal bleeding, even in the absence of initial radiographic evidence. Repeated clinical assessment, serial imaging, and timely surgical intervention are crucial for reducing the morbidity and mortality associated with this rare but potentially fatal condition.

## Introduction

Neonatal gastric perforation (NGP) is a rare but life-threatening surgical emergency that most commonly occurs during the first week of life, particularly in premature and low birth weight infants. Although its exact causes are unclear, proposed etiologies include spontaneous perforation related to congenital weakness of the gastric wall, ischemia, positive-pressure ventilation, traumatic or iatrogenic injury, and distal gastrointestinal obstruction. Early diagnosis is critical because delayed recognition is associated with rapid clinical deterioration and high mortality rates [1].

The classic presentation of NGP includes sudden abdominal distension, respiratory distress, metabolic instability, and radiographic evidence of pneumoperitoneum on abdominal imaging. Gastrointestinal bleeding, such as hematemesis or melena, is an uncommon initial manifestation and may delay diagnosis because it is more frequently attributed to other neonatal gastrointestinal disorders. Consequently, NGP may not be considered until perforation and peritonitis have progressed, particularly when initial imaging fails to demonstrate free intraperitoneal air.

This case highlights an unusual clinical presentation of a preterm, low birth weight infant who developed hematemesis and melena before the diagnosis of gastric perforation, despite an initially negative abdominal radiograph. By describing the diagnostic challenges, clinical progression, surgical management, and postoperative course, this report emphasizes the importance of maintaining a high index of suspicion and performing repeated clinical and radiologic assessments in deteriorating neonates to facilitate timely surgical intervention and improve outcomes [2].

## Case presentation

A four-day-old preterm female infant was admitted to the Neonatal Intensive Care Unit (NICU) because of prematurity, low birth weight, and respiratory distress syndrome. She was born at 31 weeks and five days of gestation via spontaneous vaginal delivery at home, with a birth weight of 1.590 kg. The APGAR score was unavailable because the delivery occurred outside the hospital; according to the parents, the infant cried immediately after birth [3].

The mother was a 37-year-old gravida 5, para 4. Maternal serologic screening for rubella, herpes simplex virus, human immunodeficiency virus (HIV), and syphilis was unavailable because prenatal care had not been received at our hospital. Laboratory investigations and newborn screening performed upon admission are summarized below (Table 1).

**Table 1:** Laboratory findings on admission. Hb: hemoglobin; RBC: red blood cell count; Platelets; TSH: thyroid-stimulating hormone; CRP: C-reactive protein; FT4: free thyroxine; FT3: triiodothyronine; sickling: abnormal hemoglobin S; G6PD: glucose-6-phosphate dehydrogenase; CCHD: critical congenital heart defects.

Parameter	Result	Reference range
Hemoglobin	144 g/L	100-140 g/L
RBC	5.13 × 10¹²/L	4.0-5.2 × 10¹²/L
WBC	8.28 × 10⁹/L	4.5-13.5 × 10⁹/L
Platelets	138 × 10⁹/L	150-450 × 10⁹/L
CRP	6.62 mg/L	0-10 mg/L
TSH (uIU/ml)	58.6 uIU/ml	0.27-4.2 uIU/ml
FT4	21.2 pmol/l	12-22 pmol/l
FT3	6.98 pmol/l	3.1-6.8 pmol/l
Sickling	Negative	Not detected
G6PD	Negative	Not detected
CCHD	Negative	Not detected

On admission to the NICU, an orogastric tube was inserted for feeding, and respiratory support was initiated with nasal continuous positive airway pressure (CPAP). The infant was subsequently weaned to 21% oxygen delivered via blender at 2 L/min. On day four of life, fresh blood was aspirated through the orogastric tube, followed by melena. Enteral feeding was discontinued, and the infant was kept nil per os (NPO). Intravenous vitamin K, fresh frozen plasma (FFP), and packed red blood cell (PRBC) transfusions were administered, and pediatric surgery was consulted.

Physical examination revealed a soft, non-distended abdomen with fresh blood in the orogastric tube and melena. An abdominal radiograph showed no evidence of pneumoperitoneum or necrotizing enterocolitis. Given the absence of radiographic evidence of perforation, the initial differential diagnosis included stress-related gastric mucosal bleeding, coagulopathy, traumatic injury from the orogastric tube, sepsis-associated gastrointestinal bleeding, necrotizing enterocolitis, or spontaneous intestinal perforation.

Over the following four hours, the infant developed progressive hematemesis and melena with rapid clinical deterioration. Given the patient's clinical presentation, repeat laboratory testing was performed (Table 2). Capillary blood gas analysis demonstrated severe metabolic acidosis (pH 7.203, PCO₂ 53 mmHg, HCO₃ 20.5 mmol/L, lactate 9 mmol/L), accompanied by significant anemia (hemoglobin 72.1 g/L and hematocrit 22.6%). Her respiratory rate was 50 breaths/minute. On reassessment, she appeared pale with poor peripheral perfusion and a mildly distended, soft abdomen.

**Table 2:** Repeat complete blood count, venous blood gases, and electrolyte panel. Hb: hemoglobin; RBC: red blood cell count; WBC: white blood cell count; pH: potential of hydrogen; PCO₂: partial pressure of carbon dioxide; HCO₃: bicarbonate; Na: sodium; K: potassium.

Parameter	Result	Reference range
Hemoglobin	72.1 g/L	100-140 g/L
RBC	5.13 × 10¹²/L	4.0-5.2 × 10¹²/L
WBC	8.28 × 10⁹/L	4.5-13.5 × 10⁹/L
Platelets	138 × 10⁹/L	150-450 × 10⁹/L
Hematocrit	22.60%	45-65%
PH	7.2	7.35-7.45
PCO₂	53 mmHg	27-41 mmHg
HCO₃	20.5 mmol/L	20-24 mmol/L
Lactate	9 mmol/L	1.1-3.5 mmol/L
Na	135.9	135-145 mmol/L
K	3.36	3.5-5.0 mmol/L

Despite aggressive resuscitation with FFP, cryoprecipitate, blood products, and vasopressor support, the infant continued to deteriorate clinically. Because of persistent gastrointestinal bleeding, worsening hemodynamic instability, and metabolic acidosis, emergency exploratory laparotomy was performed.

At surgery, the stomach was found to be significantly distended with blood clots. An anterior prepyloric gastrotomy was performed, and the blood clots were evacuated. After gastric decompression, a large perforation with friable, actively bleeding edges was identified along the greater curvature and repaired using interrupted 4-0 Maxon sutures reinforced with Lambert sutures. A 6-Fr gastrostomy tube (Feedy Feeding Tube, Romsons, Noida, India) was inserted through the initial anterior gastrotomy for gastric decompression.

Postoperatively, the infant remained mechanically ventilated, was maintained NPO, and received total parenteral nutrition together with intravenous cloxacillin, amikacin, and metronidazole. Hyponatremia, hypokalemia, and thrombocytopenia developed and were corrected with PRBCs, platelets, FFP, cryoprecipitate, tranexamic acid, and hypertonic saline.

**Figure 1:**
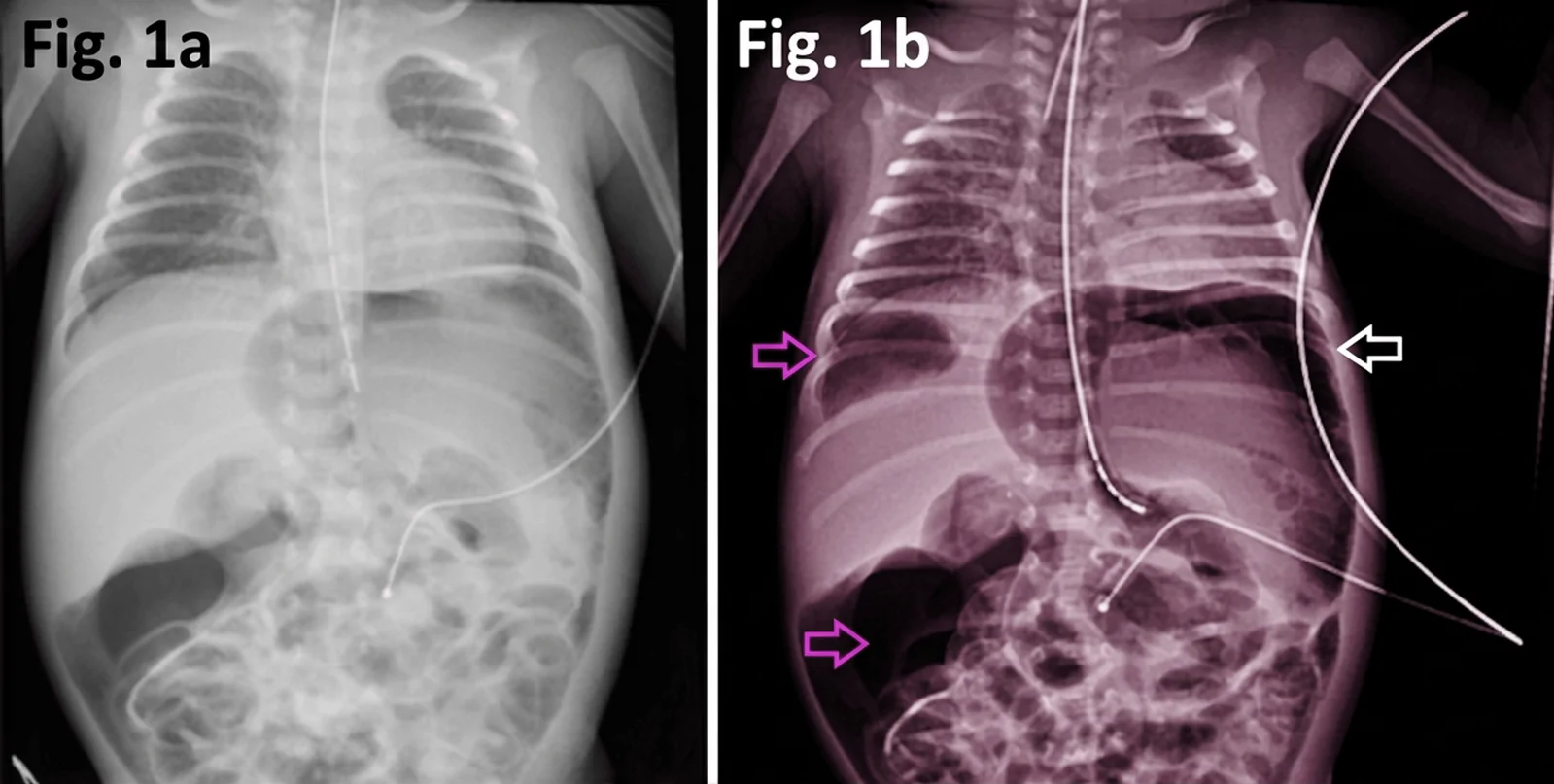
An abdominal X-ray showing sudden recurrence of significant pneumoperitoneum. (a) Description: anterior-posterior (AP) supine X-ray of the chest and abdomen of an infant. Shows various support tubes in situ and a general overview of the anatomy. A small gas bubble is seen under the left diaphragm, which is common and often benign (gastric bubble), contrasting with the diffuse appearance of pathology. (b) Description (key findings): enhanced AP supine X-ray demonstrating clear signs of pneumoperitoneum (free intraperitoneal air). Key areas are highlighted: a pronounced collection of free gas outlining the inferior surface of the diaphragm on both left and right sides, and a visible layer of air between the liver and abdominal wall, consistent with intestinal perforation.

On postoperative day four, the infant developed acute abdominal distension. Repeat abdominal radiography demonstrated pneumoperitoneum (Figure 1), raising suspicion of a postoperative gastric leak or recurrent gastric perforation. An upper gastrointestinal contrast study confirmed leakage of contrast from the stomach. Emergency re-exploration identified a recurrent gastric perforation near the esophagogastric junction, which was repaired, and an 8-Fr Stamm gastrostomy tube (Polyfeed Infant Feeding Tube, Poly Medicure Ltd., Haryana, India) was placed.

A repeat contrast study performed on postoperative day nine demonstrated no evidence of leakage, allowing the gradual initiation of gastrostomy feeding, followed by a successful transition to oral feeding.

By postoperative day 28, the gastrostomy tube was removed. The infant was discharged home the following day in stable condition on oral esomeprazole. At follow-up, she was feeding well, passing stools normally, and showed no evidence of recurrent gastrointestinal symptoms or feeding intolerance.

## Discussion

Neonatal gastric perforation (NGP) is a rare but fatal condition that requires early recognition and prompt surgical management. It continues to be associated with significant morbidity and mortality, particularly among premature and low birth weight infants [4].

The pathogenesis of NGP is not completely understood and is likely multifactorial. Several risk factors have been described, including prematurity, low birth weight, perinatal hypoxia, mechanical ventilation, continuous positive airway pressure (CPAP), gastric tube placement, sepsis, and congenital abnormalities affecting the integrity of the gastric wall. Among these, prematurity and low birth weight are consistently reported as the most common associated factors [5]. In addition, increased intragastric pressure related to positive-pressure ventilation and gastric decompression tubes may contribute to gastric wall injury. In our patient, several recognized risk factors were present, including prematurity (born at 31+ weeks), low birth weight (1.59 kg), CPAP support, and orogastric tube placement.

The clinical presentation of NGP is variable but most commonly includes progressive abdominal distension, respiratory deterioration, feeding intolerance, and signs of sepsis or shock. Radiological evidence of pneumoperitoneum is considered a classic finding and is present in the majority of reported cases. However, the diagnosis may be delayed when typical radiological findings are absent, particularly during the early stages of perforation. Free intraperitoneal air may not be immediately apparent if the perforation is small or recent, before sufficient air accumulation occurs. Therefore, the absence of pneumoperitoneum on an initial radiograph should not completely exclude the diagnosis when clinical suspicion remains high [6].

In our case, the initial presentation was hematemesis, which is an uncommon manifestation of NGP. Although upper gastrointestinal bleeding is not the typical presentation of gastric perforation, several previously reported cases have described hematemesis as an early sign of NGP. In these cases, gastrointestinal bleeding preceded the development of more characteristic findings such as abdominal distension and radiographic evidence of perforation. This highlights the importance of considering NGP in the differential diagnosis of unexplained hematemesis in high-risk neonates, particularly premature infants.

The timing of diagnosis and intervention strongly influences the prognosis of NGP. Delayed recognition can lead to progressive peritonitis, sepsis, metabolic instability, and shock, resulting in increased mortality. Reported mortality rates remain significant, ranging between approximately 20% and 50%, with poorer outcomes observed in extremely premature infants, very low birth weight infants, and patients with delayed surgical intervention or associated systemic complications. Early recognition, appropriate resuscitation, and timely surgical repair are therefore essential components of management.

The most frequent site of perforation is the greater curvature of the stomach, although other locations have also been reported. Surgical treatment typically involves primary repair of the perforation (gastrorrhaphy), with or without gastrostomy, depending on the clinical situation. Postoperative complications, including gastric leakage and infection, have been described and require close monitoring and supportive care [7].

This case highlights an unusual presentation of NGP in a preterm neonate initially presenting with hematemesis rather than the classical findings of abdominal distension and pneumoperitoneum. Although rare, NGP should remain an important diagnostic consideration in premature infants with upper gastrointestinal bleeding, especially when accompanied by risk factors such as low birth weight, respiratory support, or gastric tube placement. Maintaining a high index of suspicion may facilitate earlier diagnosis and intervention, which are critical for improving outcomes.

## Conclusions

This case highlights the importance of early recognition and prompt surgical management of neonatal gastric perforation, particularly in premature and low-birth-weight infants who may deteriorate rapidly. While neonatal gastric perforation usually presents with abdominal distension, respiratory distress, and signs of sepsis or shock, our patient presented with a rare initial manifestation of upper gastrointestinal bleeding.

The laboratory results obtained at the time of presentation showed elevated inflammatory markers, including C-reactive protein (6.62 mg/L). These findings were consistent with an inflammatory response; however, they are not specific for neonatal gastric perforation. The elevated thyroid-stimulating hormone (TSH) level (58.6 µIU/mL) was also noted, but its relationship to the gastric perforation or acute physiological stress cannot be confirmed. Therefore, this finding should be interpreted cautiously.

Due to the rarity of neonatal gastric perforation presenting with hematemesis, this case adds to the limited literature and highlights the wide range of possible clinical presentations. Recognition of unusual symptoms and timely intervention remain essential to improve outcomes in affected neonates.

## References

[1] Marrie V, Moosa A, Cywes S (1978). Gastric perforation in the newborn. S Afr Med J.

[2] Jawad AJ, Al-Rabie A, Hadi A (2002). Spontaneous neonatal gastric perforation. Pediatr Surg Int.

[3] Simon LV, Shah M, Bragg BN (2024). APGAR score. StatPearls (Internet).

[4] Garge SS, Paliwal G (2020). Neonatal gastric perforation: our experience and important preoperative and intraoperative caveats to prognosticate and improve survival. J Indian Assoc Pediatr Surg.

[5] Chen TY, Liu HK, Yang MC (2018). Neonatal gastric perforation: a report of two cases and a systematic review. Medicine (Baltimore).

[6] Huerta CT, Perez EA (2022). Diagnosis and management of neonatal gastric perforation: a narrative review. Dig Med Res.

[7] Theodorou CM, Chen P, Vanover MA, Saadai P, Brown EG, Haas KB, Hirose S (2020). Total gastrectomy with delayed Hunt-Lawrence pouch reconstruction for neonatal gastric perforation presenting with hematemesis. J Pediatr Surg Case Rep.

